# Supplementary material for: RORγt inverse agonist TF-S14 inhibits Th17 cytokines and prolongs skin allograft survival in sensitized mice
Source: Commun Biol. 2024 Apr 12;7:454. doi: 10.1038/s42003-024-06144-2 (PMC11014929; doi:10.1038/s42003-024-06144-2)
Supplement: Supplementary file 2 — Supplementary Information [file 42003_2024_6144_MOESM2_ESM.docx]

*Supplementary Information*

RORγt inverse agonist TF-S14 inhibits Th17 cytokines and prolongs skin allograft survival in sensitized mice

Ahmed Fouda^1,2,3*^, Mohamed Taoubane Maallah^1,2,3^, Araz Kouyoumdjian^2,3^, Sarita Negi^2^, Steven Paraskevas^1,2,3,4^, Jean Tchervenkov^1,2,3,4*^.

^1^Division of Surgical and Interventional Sciences, Department of Surgery, McGill University, Montréal, Québec, H3G 1A4, Canada; ^2^Research Institute of the McGill University Health Centre, Montréal, Québec, H3H 2R9, Canada; ^3^McGill University Health Centre, Montréal, Québec, H4A 3J1, Canada; ^4^Division of General Surgery, Department of Surgery, McGill University, Montréal, Québec, H3G 1A4, Canada.

***Corresponding authors:** [**ahmed.fouda@mail.mcgill.ca**](mailto:ahmed.fouda@mail.mcgill.ca)**,** [**jean.tchervenkov@muhc.mcgill.ca**](mailto:jean.tchervenkov@muhc.mcgill.ca)


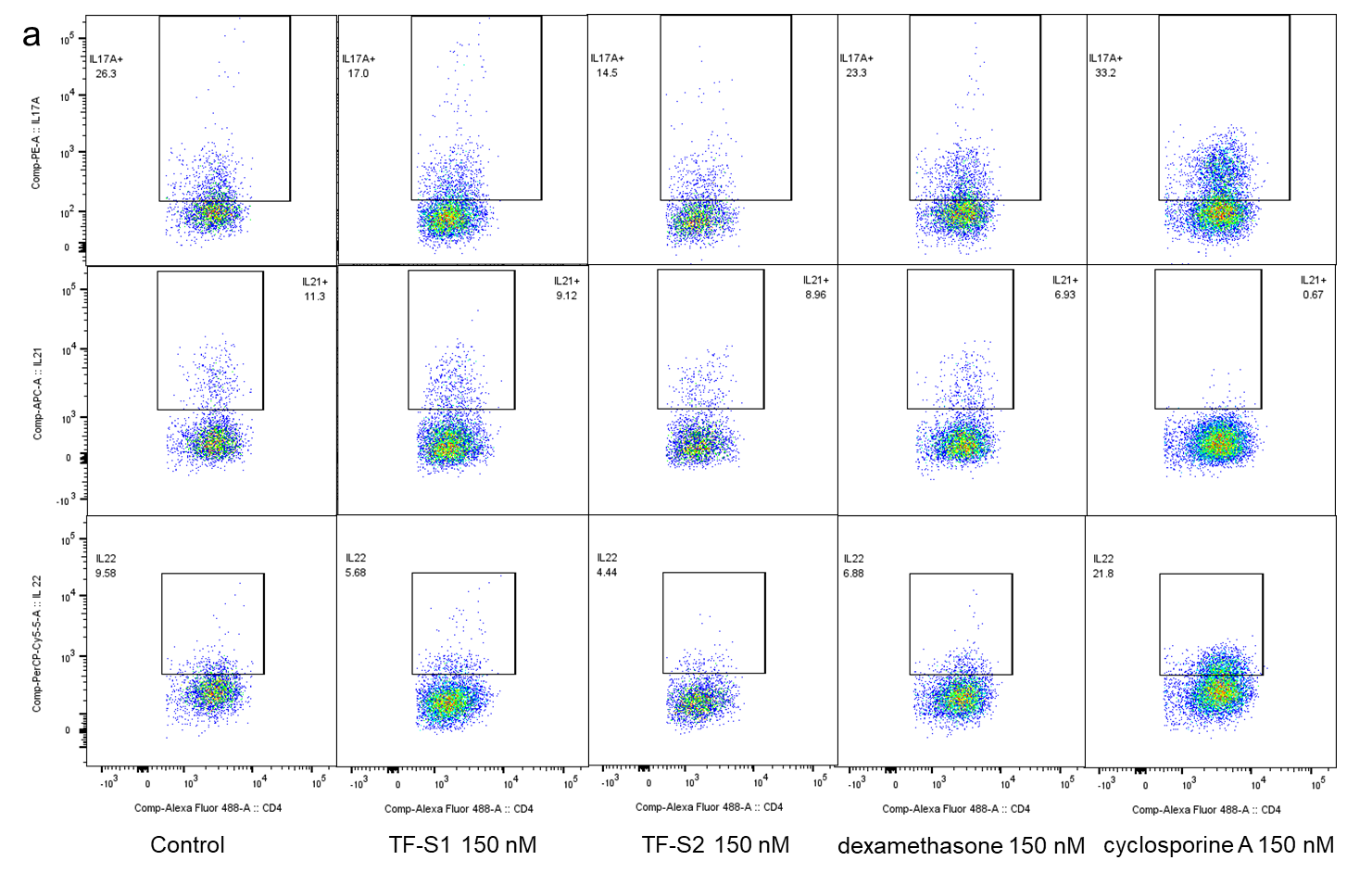


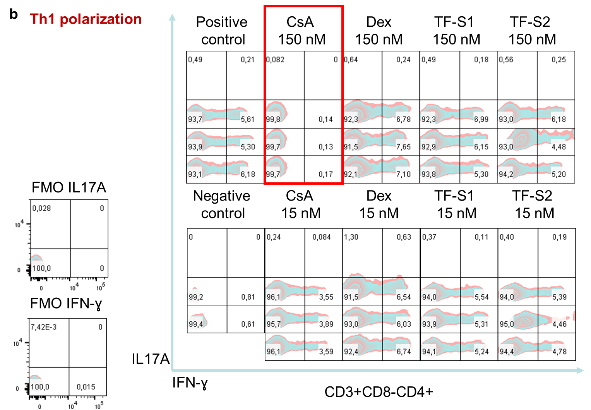

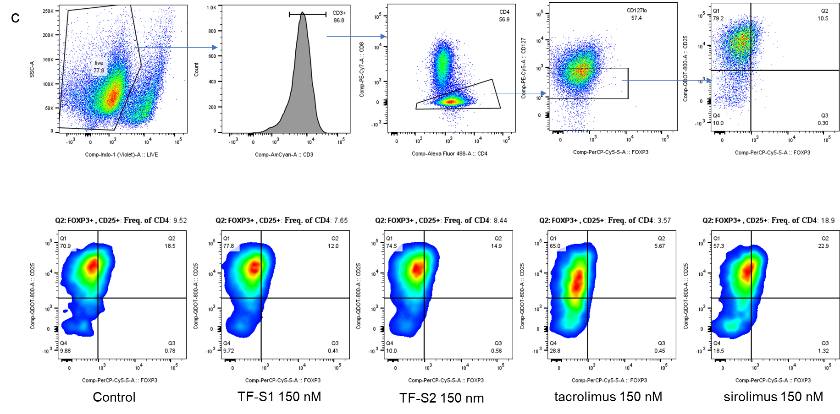


**Supplementary Fig. 1:** Representative flow cytometry data showing the effect of TF-S1, TF-S2, cyclosporine A and dexamethasone on human PBMCs Th17 polarization: the percentages of IL17A+/CD4, IL21+/CD4, IL22+/CD4 cells are shown in dot plots (a) and Th1 polarization (b); Effect of TF-S1, TF-S2, sirolimus and tacrolimus on human PBMCs Treg polarization: the gating strategy and the percentage of CD127^lo^CD25^hi^FOXP3+/CD4+ are shown in the plots and Tregs gate frequency of CD4+ cells (c).


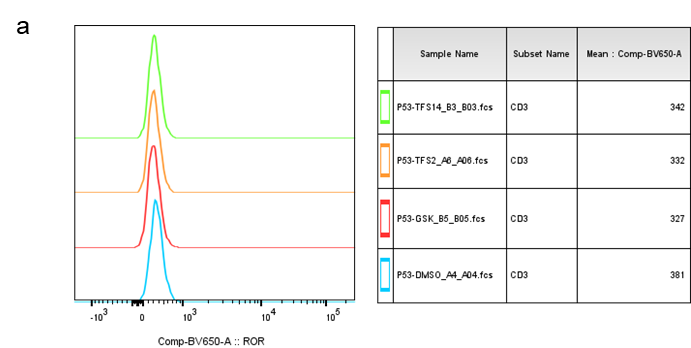


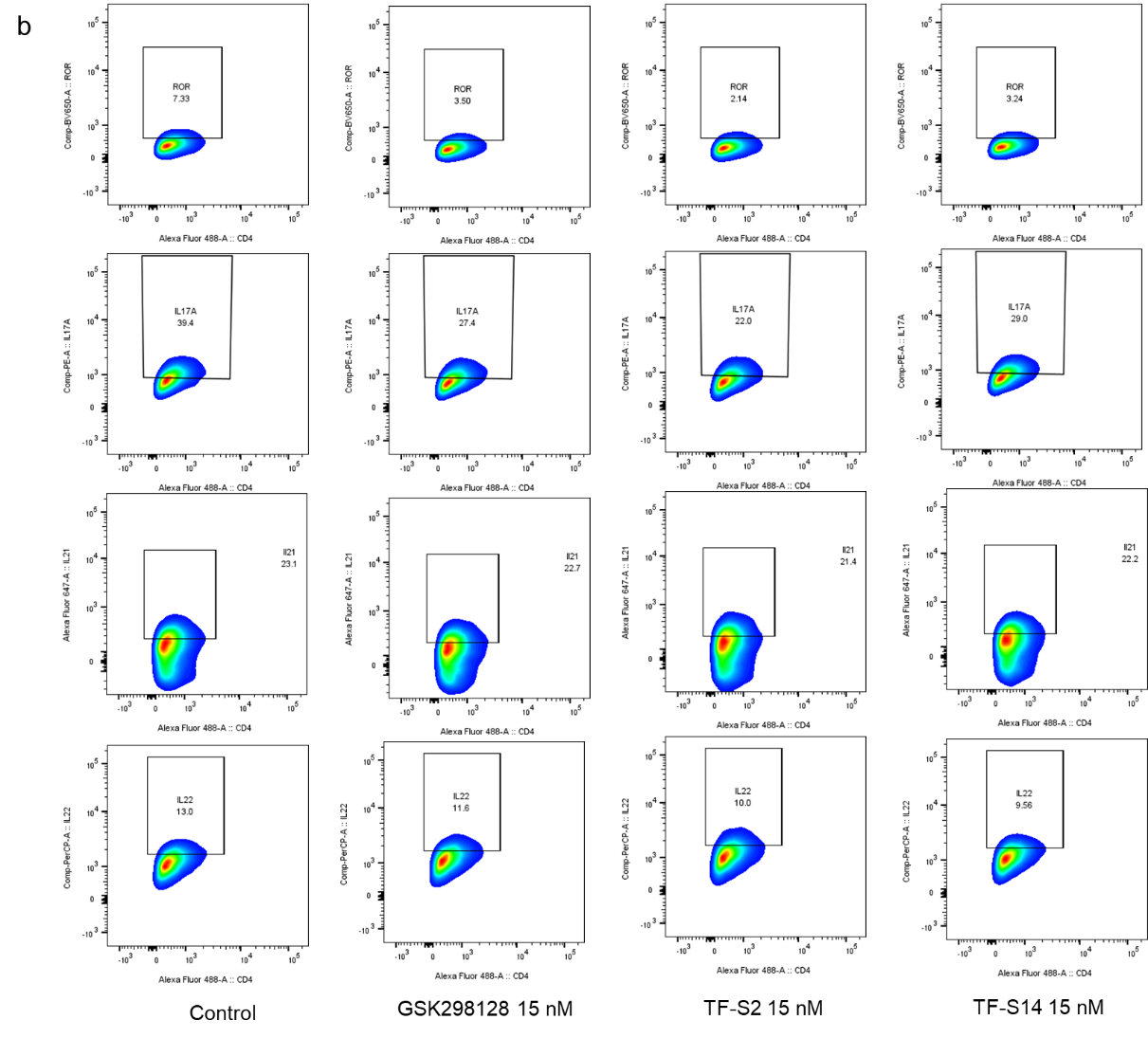


**Supplementary Fig. 2:** Representative flowcytometry plots of Th17 polarized human PBMCs of one highly sensitized transplantation candidate showing the effect of TF-S2, TF-S14 and GSK298128 on PBMCs Th17 polarization (n=7): MFI of RORγt of CD4+ are shown in flowcytometry plots and table (a). the percentages of IL17A+/CD4, IL21+/CD4, IL22+/CD4 and RORγt+/CD4 cells are shown in dot plots (b).


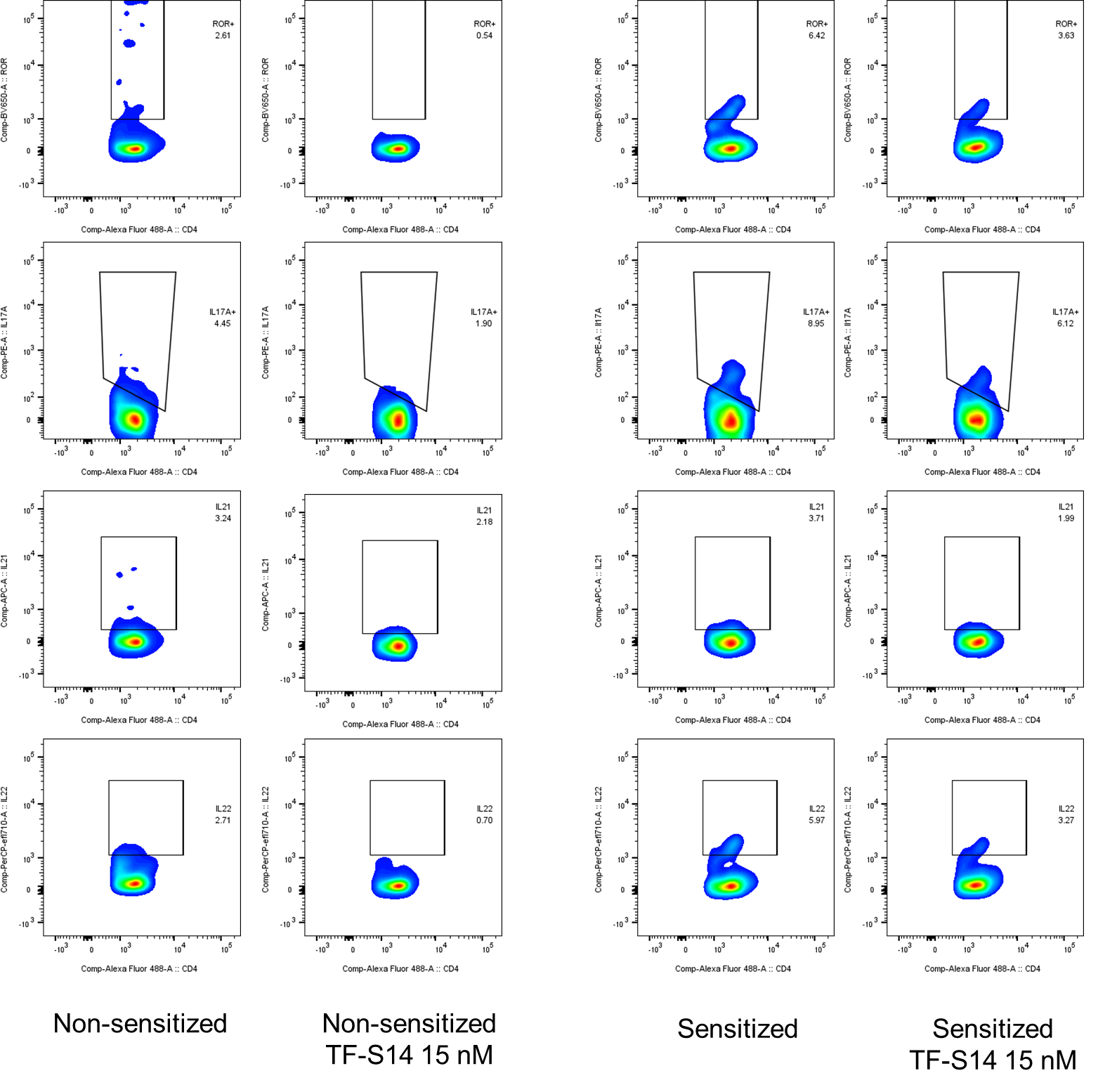


**Supplementary Fig. 3:** Representative flow cytometry data showing the effect of TF-S14 on Th17 polarization of splenocytes mouse T cells from non-sensitized and sensitized mice: the percentages of IL17A+/CD4, IL21+/CD4, IL22+/CD4 and RORγt+/CD4 cells are shown in dot plots.


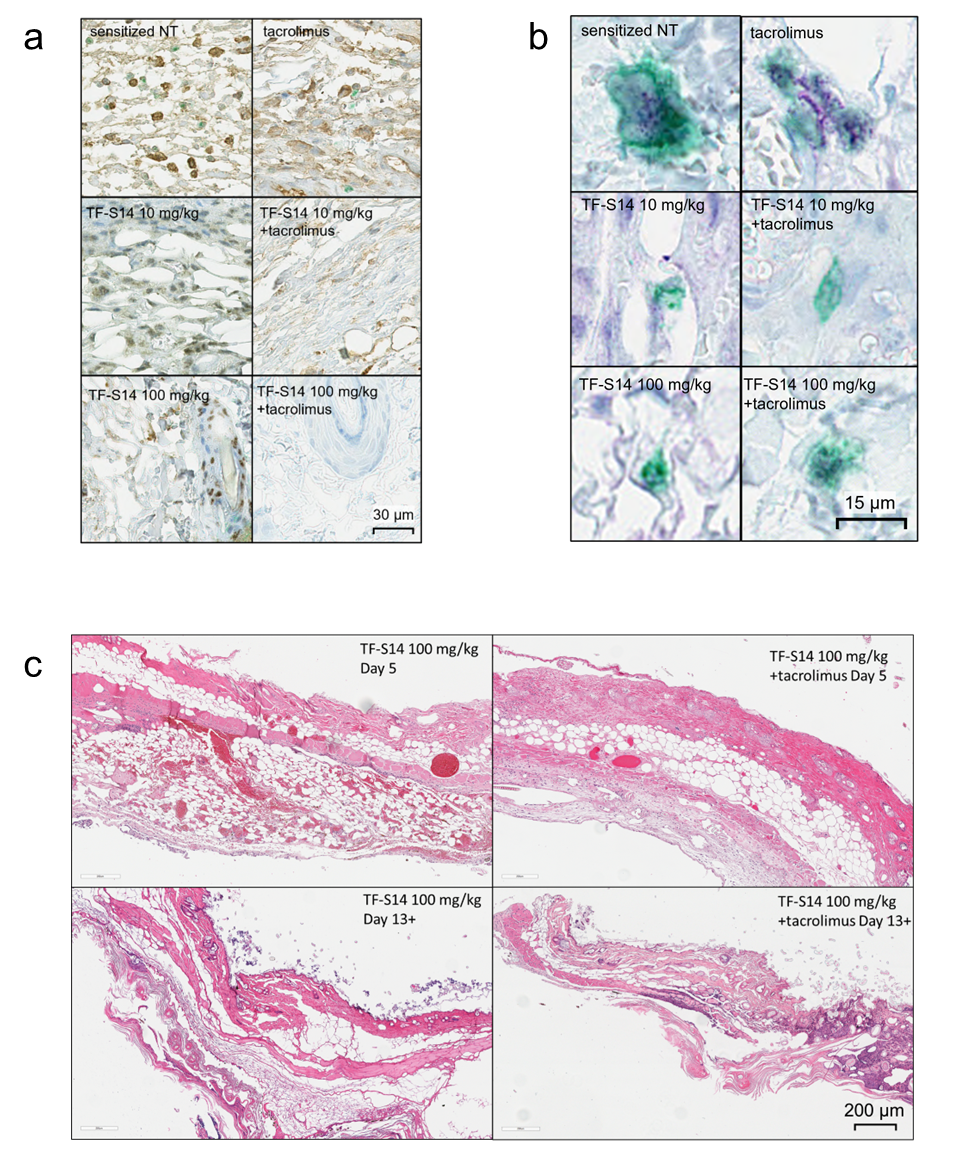


**Supplementary Fig. 4:** Dual staining of IL17A+ cells (brown) and Ly6G+ cells (green) showing Il17A+ lymphocytes in proximity of Ly6G+ neutrophils in NT and tacrolimus 0.5mg/kg only treated sensitized mice. TF-S14 treated mice show intact skin morphology and no neutrophilic or lymphocytic infiltrations (a). Dual staining of CD4+ cells (green) and IL17A+ (violet) cells showing dual stained Th17 (CD4+IL17A+) cells. Th17 cells in NT and tacrolimus only treated sensitized mice are larger in size (diameter is >10 μm) compared to cells in TF-S14 treated groups (diameter is <6 μm). Th17 cells in NT and tacrolimus only treated sensitized mice show denser granules of IL17A compared to the light-colored granules in TF-S14 treated mice (b). In TF-S14 + tacrolimus treated mice, the skin grafts that survived beyond 13 days showed thinning and fibrosis, a histological image the resembles Th2 mediated scleroderma or eosinophilic fasciitis (c).

**Supplementary Table 1.** The antibodies used in flowcytometry staining for human PBMCs.

| **antigen** | **conjugate** | **channel** | **laser** | **supplier** | **catalogue no.** |
| --- | --- | --- | --- | --- | --- |
| CD3 | BV510 | Amcyan | violet | Biolegend | 300447 |
| CD4 | Alexa Fluor 488 | FITC | blue | Life technologies | 11-0048-41 |
| CD8 | PE-Cy7 | PE-Cy7 | yellow | Life technologies | 25-0084-82 |
| CD25 | BV786 | Qdot800 | violet | BD biosciences | 563701 |
| CD127 | PE-Cy5 | PE-Cy5 | Yellow | Life technologies | 15-1278-42 |
| FOXP3 | PerCP-Cy5.5 | PerCP-Cy5.5 | blue | BD biosciences | 561493 |
| IFNγ | APC-eFluor 780 | APC-Cy7 | red | Life technologies | 47-7319-42 |
| IL17A | PE | PE | yellow | BD biosciences | 560487 |
| IL21 | APC | APC | red | BD biosciences | 560493 |
| IL22 | PerCP-eFluor 710 | PerCP-Cy5.5 | blue | Life technologies | 46-7229-42 |
| RORγt | BV650 | Qdot655 | violet | BD biosciences | 563424 |
| Viability fixable dye | eFluor 455 (UV) | Indo-1 (violet) | UV | Life technologies | 65-0868-14 |

**Supplementary Table 2.** The antibodies used in the flowcytometry staining for mouse splenocytes.

| **antigen** | **conjugate** | **channel** | **laser** | **supplier** | **catalogue no.** |
| --- | --- | --- | --- | --- | --- |
| CD3 | BV510 | Amcyan | violet | Biolegend | 100233 |
| CD4 | Alexa Fluor 488 | FITC | blue | Life technologies | 53-0041-82 |
| CD8 | PE-Cy7 | PE-Cy7 | yellow | Life technologies | 25-0081-81 |
| CD45 | APC-Cy7 | APC-Cy7 | red | BD biosciences | 561037 |
| CD117 | APC | APC | red | BD biosciences | 561074 |
| CD127 | PerCP-eFluor 710 | PerCP-Cy5.5 | blue | Life technologies | 46-1271-80 |
| CD138 | PerCP-Cy5.5 | PerCP-Cy5.5 |  |  |  |
| CD169 | FITC | FITC | blue | Life technologies | MA5-28189 |
| IFNγ | APC-eFluor 780 | APC-Cy7 | red | Life technologies | 47-7311-80 |
| IgG3 | Alexa Fluor 594 | Texas red | yellow | BD biosciences | A-21155 |
| IgM | Alexa Fluor 350 | Indo-1 (violet) | UV | Life technologies | A-31552 |
| IL17A | PE | PE | yellow | Life technologies | 12-7177-81 |
| IL21 | APC | APC | red | Life technologies | 17-7211-80 |
| IL22 | PerCP-eFluor 710 | PerCP-Cy5.5 | blue | Life technologies | 46-7221-80 |
| Linage (Lin) | eFluor 450 | Alexa Fluor 405 | violet | Life technologies | 88-7772-72 |
| Ly6G | FITC | FITC | blue | Life technologies | 11-9668-80 |
| RORγt | BV650 | Qdot655 | violet | BD biosciences | 564722 |
| Viability fixable dye | eFluor 455 (UV) | Indo-1 (violet) | UV | Life technologies | 65-0868-14 |
| Viability fixable dye | eFluor 506 | Amcyan | violet | Life technologies | 65-0866-14 |
| Viability  fixable dye | eFluor 780 | APC-Cy7 | red | Life technologies | 65-0865-14 |

**Supplementary Table 3.** The antibodies used for immune fluorescence staining of mouse spleen sections.

| **anitgen** | **excitation** | **emission** | **supplier** | **catalogue no** |
| --- | --- | --- | --- | --- |
| IgM | 343 nm | 441 nm | Life technologies | A-31552 |
| IgG | 590 nm | 618 nm | Life technologies | A-21155 |
| Ly6G | 498 nm | 517 nm | Life technologies | 11-9668-80 |

**Supplementary Table 4.** The antibodies used in immunohistochemistry for mouse skin sections.

| **antigen** | **supplier** | **catalogue no** |
| --- | --- | --- |
| CD4 | Santa Cruz | sc-19641 |
| IL17A | R&D Systems | AF317-NA |
| Ly6G | BD biosciences | 551459 |

**Supplementary Table 5.** The cytokines used in human PBMCs polarization.

| **cytokine** | **source** | **supplier** | **catalogue no.** |
| --- | --- | --- | --- |
| IL1β | E. coli | Peprotech | 200-01B-2UG |
| IL2 | E. coli | Peprotech | 200-02-10UG |
| IL6 | E. coli | Peprotech | 200-06-5UG |
| IL23 | Insect cells | Peprotech | 200-23-2UG |
| TGFβ1 | HEK293 | Peprotech | 100-21-2UG |

**Supplementary Table 6.** The cytokines used in mouse splenocytes Th17 polarization.

| **cytokine** | **source** | **supplier** | **catalogue no.** |
| --- | --- | --- | --- |
| IL1β | E. coli | Peprotech | 211-11B-2UG |
| IL6 | E. coli | Peprotech | 216-16-2UG |
| IL23 | Insect cells | Life technologies | 14-8231-63 |
| TGFβ1 | HEK293 | Peprotech | 100-21-2UG |

**Supplementary Table 7.** Other materials used in human PBMCs polarization experiments.

| **Item** | **supplier** | **catalogue no.** |
| --- | --- | --- |
| Cell stimulation cocktail | Life technologies | 00-4975-93 |
| Human CD3/CD28 dynabeads | Life technologies | 11161D |
| Human IL-17A ELISA | Biolegend | 433914 |
| Human IL21 ELISA | Biolegend | 433804 |
| GSK2981278 | Cayman chemicals | 20974-1 |
| Rapamycin (Sirolimus) | ApexBio | A8167-5 |
| Tacrolimus | MP biomedicals | 0218311610 |

**Supplementary Table 8.** Other materials used in mouse experiments.

| **Item** | **supplier** | **catalogue no.** |
| --- | --- | --- |
| Cell stimulation cocktail | Life technologies | 00-4975-93 |
| Easysep mouse T Cell isolation kit | Stemcell | 19851 |
| Mouse CD3/CD28 dynabeads | Life technologies | 11456D |
| IgG (total) mouse ELISA kit | Life technologies | 88-50400-22 |
| IgG3 mouse ELISA kit | Life technologies | 88-50440-22 |
