# Supplementary material for: RORγt inverse agonist TF-S14 inhibits Th17 cytokines and prolongs skin allograft survival in sensitized mice
Source: Commun Biol. 2024 Apr 12;7:454. doi: 10.1038/s42003-024-06144-2 (PMC11014929; doi:10.1038/s42003-024-06144-2)
Supplement: Supplementary file 3 — Description of Additional Supplementary Files [file 42003_2024_6144_MOESM3_ESM.docx]

**Description of Additional Supplementary Files**

File name: Supplementary Data 1

Description: Numerical source data for graphs and charts in Figure 1.

File name: Supplementary Data 2

Description: Numerical source data for graphs and charts in Figure 2.

File name: Supplementary Data 3

Description: Numerical source data for graphs and charts in Figure 3.

File name: Supplementary Data 4

Description: Numerical source data for graphs and charts in Figure 4.

File name: Supplementary Data 5

Description: Numerical source data for graphs and charts in Figure 5.
