# Supplementary material for: RORγt inverse agonist TF-S14 inhibits Th17 cytokines and prolongs skin allograft survival in sensitized mice
Source: Commun Biol. 2024 Apr 12;7:454. doi: 10.1038/s42003-024-06144-2 (PMC11014929; doi:10.1038/s42003-024-06144-2)
Supplement: Supplementary file 9 — Reporting Summary [file 42003_2024_6144_MOESM9_ESM.pdf]

nature portfolio

Corresponding author(s): Ahmed Fouda

Last updated by author(s): Feb 13, 2024

Reporting Summary

Nature Portfolio wishes to improve the reproducibility of the work that we publish. This form provides structure for consistency and transparency in reporting. For further information on Nature Portfolio policies, see our Editorial Policies and the Editorial Policy Checklist.

Statistics

For all statistical analyses, confirm that the following items are present in the figure legend, table legend, main text, or Methods section.

n/a	Confirmed	
☐	☒	The exact sample size (*n*) for each experimental group/condition, given as a discrete number and unit of measurement
☐	☒	A statement on whether measurements were taken from distinct samples or whether the same sample was measured repeatedly
☐	☒	The statistical test(s) used AND whether they are one- or two-sided<br>*Only common tests should be described solely by name; describe more complex techniques in the Methods section.*
☐	☒	A description of all covariates tested
☐	☒	A description of any assumptions or corrections, such as tests of normality and adjustment for multiple comparisons
☐	☒	A full description of the statistical parameters including central tendency (e.g. means) or other basic estimates (e.g. regression coefficient) AND variation (e.g. standard deviation) or associated estimates of uncertainty (e.g. confidence intervals)
☐	☒	For null hypothesis testing, the test statistic (e.g. *F*, *t*, *r*) with confidence intervals, effect sizes, degrees of freedom and *P* value noted<br>*Give P values as exact values whenever suitable.*
☐	☒	For Bayesian analysis, information on the choice of priors and Markov chain Monte Carlo settings
☐	☒	For hierarchical and complex designs, identification of the appropriate level for tests and full reporting of outcomes
☐	☒	Estimates of effect sizes (e.g. Cohen's *d*, Pearson's *r*), indicating how they were calculated

Our web collection on statistics for biologists contains articles on many of the points above.

Software and code

Policy information about availability of computer code

Data collection	BD FACSDiva Software (flow cytometry), ZEISS ZEN lite (immunofluresence)
Data analysis	graphpad prism (statistical analysis), flowjo (flow cytometry), ImageJ, (immunofluresence MFI), Aperio Imagescope (Immunohistochemistry scoreing)

For manuscripts utilizing custom algorithms or software that are central to the research but not yet described in published literature, software must be made available to editors and reviewers. We strongly encourage code deposition in a community repository (e.g. GitHub). See the Nature Portfolio guidelines for submitting code & software for further information.

Data

Policy information about availability of data

All manuscripts must include a data availability statement. This statement should provide the following information, where applicable:

- Accession codes, unique identifiers, or web links for publicly available datasets
- A description of any restrictions on data availability
- For clinical datasets or third party data, please ensure that the statement adheres to our policy

N/A

Research involving human participants, their data, or biological material

Policy information about studies with human participants or human data. See also policy information about sex, gender (identity/presentation), and sexual orientation and race, ethnicity and racism.

Reporting on sex and gender	N/A
Reporting on race, ethnicity, or other socially relevant groupings	N/A
Population characteristics	N/A
Recruitment	N/A
Ethics oversight	McGill University (animal ethics committee) and McGill University Health Centre (approved for collections of blood from healthy volunteers and transplantation patients)

Note that full information on the approval of the study protocol must also be provided in the manuscript.

Field-specific reporting

Please select the one below that is the best fit for your research. If you are not sure, read the appropriate sections before making your selection.

☒ Life sciences ☐ Behavioural & social sciences ☐ Ecological, evolutionary & environmental sciences

For a reference copy of the document with all sections, see nature.com/documents/nr-reporting-summary-flat.pdf

Life sciences study design

All studies must disclose on these points even when the disclosure is negative.

Sample size	human PBMCS samples n=7+9+3=19 for mice ~200
Data exclusions	N/A
Replication	In vitro experiments at least 2 independant repetitions per experiment an
Randomization	in animal study animals were randomly distributed into group follow the surgery
Blinding	Blinding was performed in some animal experiments . The Th17 polazrization over time the samples were blinded for prior sensitization status and cPRA

Reporting for specific materials, systems and methods

We require information from authors about some types of materials, experimental systems and methods used in many studies. Here, indicate whether each material, system or method listed is relevant to your study. If you are not sure if a list item applies to your research, read the appropriate section before selecting a response.

Materials & experimental systems

n/a	Involved in the study	
☐	☒	Antibodies
☒	☐	Eukaryotic cell lines
☒	☐	Palaeontology and archaeology
☐	☒	Animals and other organisms
☐	☒	Clinical data
☒	☐	Dual use research of concern
☒	☐	Plants

Methods

n/a	Involved in the study	
☒	☐	ChIP-seq
☐	☒	Flow cytometry
☒	☐	MRI-based neuroimaging

Antibodies

Antibodies used	The antibodies used in flowcytometry staining for human PBMCs.<br>antigen conjugate channel laser supplier catalogue no.<br>CD3 BV510 Amcyan violet Biolegend 300447

CD4 Alexa Fluor 488 FITC blue Life technologies 11-0048-41
CD8 PE-Cy7 PE-Cy7 yellow Life technologies 25-0084-82
CD25 BV786 Qdot800 violet BD biosciences 563701
CD127 PE-Cy5 PE-Cy5 Yellow Life technologies 15-1278-42
FOXP3 PerCP-Cy5.5 PerCP-Cy5.5 blue BD biosciences 561493
IFNγ APC-eFluor 780 APC-Cy7 red Life technologies 47-7319-42
IL17A PE PE yellow BD biosciences 560487
IL21 APC APC red BD biosciences 560493
IL22 PerCP-eFluor 710 PerCP-Cy5.5 blue Life technologies 46-7229-42
RORγt BV650 Qdot655 violet BD biosciences 563424
Viability fixable dye eFluor 455 (UV) Indo-1 (violet) UV Life technologies 65-0868-14

The antibodies used in the flow cytometry staining for mouse splenocytes.
antigen conjugate channel laser supplier catalogue no.
CD3 BV510 Amcyan violet Biolegend 100233
CD4 Alexa Fluor 488 FITC blue Life technologies 53-0041-82
CD8 PE-Cy7 PE-Cy7 yellow Life technologies 25-0081-81
CD45 APC-Cy7 APC-Cy7 red BD biosciences 561037
CD117 APC APC red BD biosciences 561074
CD127 PerCP-eFluor 710 PerCP-Cy5.5 blue Life technologies 46-1271-80
CD138 PerCP-Cy5.5 PerCP-Cy5.5
CD169 FITC FITC blue Life technologies MA5-28189
IFNγ APC-eFluor 780 APC-Cy7 red Life technologies 47-7311-80
IgG3 Alexa Fluor 594 Texas red yellow BD biosciences A-21155
IgM Alexa Fluor 350 Indo-1 (violet) UV Life technologies A-31552
IL17A PE PE yellow Life technologies 12-7177-81
IL21 APC APC red Life technologies 17-7211-80
IL22 PerCP-eFluor 710 PerCP-Cy5.5 blue Life technologies 46-7221-80
Linage (Lin) eFluor 450 Alexa Fluor 405 violet Life technologies 88-7772-72
Ly6G FITC FITC blue Life technologies 11-9668-80
RORγt BV650 Qdot655 violet BD biosciences 564722
Viability fixable dye eFluor 455 (UV) Indo-1 (violet) UV Life technologies 65-0868-14
Viability fixable dye eFluor 506 Amcyan violet Life technologies 65-0866-14
Viability
fixable dye eFluor 780 APC-Cy7 red Life technologies 65-0865-14

The antibodies used for immune fluorescence staining of mouse spleen sections.
anitgen excitation emission supplier catalogue no
IgM 343 nm 441 nm Life technologies A-31552
IgG 590 nm 618 nm Life technologies A-21155
Ly6G 498 nm 517 nm Life technologies 11-9668-80
The antibodies used in immunohistochemistry for mouse skin sections.
antigen supplier catalogue no
CD4 Santa Cruz sc-19641
IL17A R&D Systems AF317-NA
Ly6G BD biosciences 551459

Other materials used in human PBMCs polarization experiments.
Item supplier catalogue no.
Human CD3/CD28 dynabeads Life technologies 11161D
Human IL-17A ELISA Biolegend 433914
Human IL21 ELISA Biolegend 433804

Other materials used in mouse experiments.
Item supplier catalogue no.
Cell stimulation cocktail Life technologies 00-4975-93
Easysep mouse T Cell isolation kit Stemcell 19851Mouse CD3/CD28 dynabeads Life technologies 11456D
IgG (total) mouse ELISA kit Life technologies 88-50400-22
IgG3 mouse ELISA kit Life technologies 88-50440-22

Validation: For all antibodies validation can be accessed on manufacturer's website using the catalogue number

Animals and other research organisms

Policy information about studies involving animals; ARRIVE guidelines recommended for reporting animal research, and Sex and Gender in Research

Laboratory animals: C57BL/6 and Balb/c mice

Wild animals: N/A

Reporting on sex: N/A

Field-collected samples	Patients samples were collected from the hospital sites and processed within 1 hour of collection
Ethics oversight	McGill University and McGill University Health Centre<br>In vitro experiments using blood samples<br>animal study (animal ethics committee)

Note that full information on the approval of the study protocol must also be provided in the manuscript.

Clinical data

Policy information about clinical studies

All manuscripts should comply with the ICMJE guidelines for publication of clinical research and a completed CONSORT checklist must be included with all submissions.

Clinical trial registration	N/A
Study protocol	N/A
Data collection	N/A
Outcomes	N/A

Plants

Seed stocks	N/A
Novel plant genotypes	N/A
Authentication	N/A

Flow Cytometry

Plots

Confirm that:

☒ The axis labels state the marker and fluorochrome used (e.g. CD4-FITC).

☒ The axis scales are clearly visible. Include numbers along axes only for bottom left plot of group (a 'group' is an analysis of identical markers).

☒ All plots are contour plots with outliers or pseudocolor plots.

☒ A numerical value for number of cells or percentage (with statistics) is provided.

Methodology

Sample preparation	PBMCs, Th1, Treg, Th17 polarized PBMCs<br>Mouse splenocytes isolated activated and cytokines stained intracellularly
Instrument	BD LSR-Fortessa flow cytometer
Software	BD FACSDiva™ Software
Cell population abundance	The least cell count 15000 and up to 200000 cell per sample per acquisition
Gating strategy	Cells>single cells>live cells>CD3+>CD4+>IL17A, IL21, IL22, IFN<br>Cells>single cells>live cells>CD3+>CD4+>IL17A, IFN<br>Cells>single cells>live cells>CD3+>CD4+>CD25hi>CD127lo>Foxp3+ (gating provided)<br>mouse splenocytes<br>Cells>single cells>live cells>CD3+>CD4+>IL17A, IL21, IL22 or IFN<br>Cells>single cells>live cells>CD3->Lin->CD117+>CD127+<br>Cells>single cells>live cells>CD3->CD19+CD183+>IgM vs IgG3

☒ Tick this box to confirm that a figure exemplifying the gating strategy is provided in the Supplementary Information.
